# The Relation of Local Societies to the State Association

**Published:** 1892-08

**Authors:** 


### EDITORIAL DEPARTMENT.

### THE RELATION OF LOCAL SOCIETIES TO THE STATE ASSOCIATION.

### AN IMPORTANT DECISION.

Is the West Texas Medical Society a branch of the Texas State Medical Association? Are State medical associations branches of or subordinate bodies to the American Medical Association?

The answer to one of the above questions would seem to apply to the other, and in view of certain complications that have arisen in this connection, it is highly important that an understanding should be arrived at.

That the local societies in this State regard themselves as "branches" of the State Association,—or, at least, consider themselves subordinate to the State Association, is evidenced by the fact that nearly all local societies annually send delegates (and formerly paid tribute in money) to the State Association, and many of them in framing their constitutions and by-laws, have provided for an appeal to the State Association in certain cases. It seems to be generally understood among medical men that such is the relation of the local societies to the State Association, and of the State Association to the National organization. Especially with regard to the latter would this seem to be accepted as the proper relation; for the several State Societies have adopted the Code of Ethics of the American Medical Association, and have provided for an appeal to that body in certain matters of dispute.

But as a matter of fact,—and in the eyes of the law, does any such relation of dependence on the one hand and of authority on the other really exist?

The Texas State Medical Association is a chartered institution, and so is the American Medical Association. Those charters make no provision for issuing charters to the local or State societies, and as a matter of fact no such charters are extant. The subject has often been discussed, both in the American Medical Association and in the Texas State Association; but all propositions looking to a unification of the profession, i. e., of establishing "branch societies," we believe, have been voted down. In Great Britain all local societies are chartered by the British Medical Association and are, therefore, "branches" of the National body. The West Texas Medical Society also holds a charter from the State; and while the members acknowledge allegiance to the State Medical Association, it is, in fact, an independent organization. That is the decision recently given by Judge King* of the 45th (Texas) Judicial District Court, at San Antonio, Tex., (April 13).

We presume the decision will become a precedent and will apply to all other local societies holding charters from the State; and, inferentially, will apply also to the Texas Medical Association, which is chartered by the State.

The test came up in the matter of the expulsion of Dr. James Kennedy‡ from the West Texas Medical Society.

It will be remembered Dr. Kennedy claimed that his expulsion was illegal and not in conformity to the constitution and by-laws of the society; and that he applied to the District Court for a mandamus to compel his restoration to membership, the erasure of the entry in the record, and to prevent the West Texas Medical Society reporting his expulsion to the Texas State Medical Association, he being a member of that body. Final action has not yet been had on this application, and the West Texas Medical Society proceeded to file the information with the State Association at the meeting in Tyler in April last. It will be remembered also, that the latter body supposing it to be a "charge," (we assume they did, from the action taken in the matter) referred the papers sent up by the West Texas Medical Society to their Judicial Council, who reported that "gross injustice" had been done Dr. Kennedy, and they "could not approve the verdict," that his expulsion "was irregular," etc., and referred the matter back "for adjudication" by the West Texas Society "before an appeal could be taken to the State Medical Association," which (appeal) they said, was "Dr. Kennedy's proper course."

---

*And also by Judge Noonan, of the 37th District Court.

‡Dr. Russell Caffery, who was associated with Dr. Kennedy in the "operation" out of which grew all this trouble, and who was a member also of the West Texas Medical Society, bus not a member of the State Association, was expelled by his society at the same time Dr. Kennedy was. He applied, also, for a mandamus, and his case was tried in the 37th District Court, Judge Noonan. In this case the same decision was made as in Kennedy's case.

On April 13th proceedings were had by the 45th District Court at San Antonio on the application of Dr. Kennedy for mandamus, and the first point to be settled was the relation between the medical bodies; the West Texas Medical Society filed an answer, setting forth that the court has no jurisdiction to try the case, as Dr. Kennedy had the right to appeal to the State Medical Association from the action of the local society; that mandamus being last resort cannot issue until Kennedy had failed of relief at the hands of all intermediate tribunals, and that the State Association was the highest medical tribunal, etc. This plea was overruled by Judge King, who decided,—after having examined the constitution and by-laws of the two organizations and finding that both were chartered by the State,—that the State Medical Association is not a tribunal to which an appeal could or should be made, and that Kennedy had no right to so appeal; that therefore the court *has* jurisdiction and will proceed to try the case on its merits at a time set. The points in the trial yet to settle, being, whether or not Dr. Kennedy (and Dr. Caffery) were expelled in due process and in accordance with the provisions of the laws of the West Texas Medical Society.

In the face of this judicial decision, the question may pertinently be asked, what has the State Association to do with *any* action of the West Texas Medical Society; and by what obligations is the latter bound to heed the action of the Judicial Council had at Tyler? In an "open letter to Secretary West" (in our July number) the West Texas Medical Society declines to "correct irregularities, of the existence of which it is entirely ignorant."

Assuming that the possession of a charter from the State by a medical society, and the absence of one from a higher (?) society constitutes a medical organization an "independent body;" assuming that Judge King's and Judge Noonan's decisions will apply as well to the State Medical Association in its relation to the American Medical Association, the question as pertinently arises—What has the American Medical Association to do with any action of the Texas State Medical Association? And what obligation rests upon the latter to heed any decision of the former upon any case appealed?

True, such appeal is provided for in its by-laws, but it is stipulated that a complete transcript of the record of the proceedings of the lower court (?) shall accompany it. In a certain case to which allusion is made and which we do not care to mention more specifically, no transcript of the record was furnished, and the decision that the State Medical Association had not conformed to its own by-laws was *ex parte*, and would have no weight in any court at law.

The Texas State Medical Association, at its meeting at Galveston next May, will have to consider and determine two cases in which these points are involved, and the above is thrown out for consideration of members and those concerned.

